# The Use of Long-Acting Injectable Antipsychotics in Schizophrenia

**DOI:** 10.1007/s40501-017-0115-z

**Published:** 2017-04-19

**Authors:** Seiya Miyamoto, W. Wolfgang Fleischhacker

**Affiliations:** 1 Department of Psychiatry, Sakuragaoka Memorial Hospital, 1-1-1 Renkoji, Tama-shi, Tokyo 206-0021 Japan; 2 Department of Psychiatry, Psychotherapy and Psychosomatics, Medical University Innsbruck, Anichstrasse 35, A-6020 Innsbruck, Austria

**Keywords:** Schizophrenia, Long-acting injectable antipsychotic, Risperidone, Paliperidone palmitate, Olanzapine pamoate, Aripiprazole, Depot, Neuroleptic, Treatment

## Abstract

Schizophrenia is a mostly chronic mental disorder, and symptomatic relapse is frequently observed. It is often associated with social and/or occupational decline that can be difficult to reverse. Most patients with the illness need long-term pharmacological treatment, and antipsychotic drugs represent the mainstay of clinical care. Long-acting injectable antipsychotics (LAIs) are an important alternative to oral medication, particularly advantageous in the context of compliance management. Several new-generation antipsychotics (NGAs), including risperidone, olanzapine, paliperidone, and aripiprazole, have become available as long-acting formulations, and new evidence has been accumulating. To date, all of the NGA LAIs have demonstrated a statistically and clinically significant decrease of relapse rates over placebo. The results of clinical trials comparing NGA LAIs with oral antipsychotics (OAPs) are not consistent, as being influenced considerably by study design. Superiority of LAIs to OAPs in efficacy is most evident in mirror image and cohort studies. New-generation LAIs are comparable to their oral mother compounds regarding safety and tolerability if one disregards potential injection site complications. There is little evidence of efficacy differences between the available LAIs, but they have different characteristics in terms of pharmacodynamic and pharmacokinetic profiles, injection interval, cost, requirements for oral supplementation, as well as adverse events. Considering these differences is useful for selecting LAIs for the treatment of individual patients. There is increasing evidence suggesting the use of LAIs in special patient groups, such as first-episode or forensic schizophrenia patients. This article reviews data on the use of NGA LAIs in schizophrenia and discusses current issues from clinical and methodological perspectives.

## Introduction

Schizophrenia is a mostly chronic mental disorder, and symptomatic relapse is frequently observed during the course of the illness. It is often associated with social and/or occupational decline that can be difficult to reverse. Most patients with schizophrenia need long-term pharmacological treatment, and antipsychotic drugs represent the mainstay of clinical care [1]. Long-acting injectable antipsychotics (LAIs) were developed in the 1960s and were originally thought to be particularly advantageous for patients with adherence problems and for those with a history of severe relapse upon medication discontinuation [2]. The use of first-generation antipsychotic (FGA) LAIs has recently decreased since several new-generation antipsychotics (NGAs), including risperidone, olanzapine, paliperidone, and aripiprazole, have become available as long-acting formulations and new evidence has been accumulating. This article updates the information on the use of NGA LAIs in schizophrenia and discusses current issues from clinical perspectives. Information on the results of placebo-controlled registration trials of NGA LAIs is not detailed in this review, because all of them have demonstrated a significant positive impact on relapse rates and symptoms over placebo in patients with chronic schizophrenia as well as in acutely ill patients [3, 6••].

## General considerations

Here, we provide considerations pertaining to all available agents. More specific findings concerning the individual drugs are reviewed in the respective chapters.

## Efficacy of LAIs versus oral antipsychotics

Meta-analyses of randomized controlled trials (RCTs) comparing NGA LAIs with either placebo or oral antipsychotics (OAPs) have provided discrepant results. While NGA LAIs have consistently demonstrated greater efficacy than placebo, significant differences compared with OAPs are much less evident [5, 7–9]. However, RCTs might not be the best strategy to evaluate the efficacy of LAIs because of selective recruitment as well as indirect compliance enhancing measures, such as frequent visits, and increased encouragement to stay in studies and thereby on treatment [4, 10, 11].

Mirror image studies comparing periods of treatment with LAIs versus OAPs in the same patients might better reflect the impact of LAIs in a real-world setting [11]. A meta-analysis of 25 mirror image studies of LAIs (*n* = 5940) demonstrated superiority of LAIs over OAPs in preventing a next hospitalization [11]. Cohort studies show mixed results, but most of them report better outcomes for LAIs than for OAPs [10, 12–14]. In a meta-analysis that included 58 studies with both interventional and non-interventional designs, using meta-regression analyses, LAIs were superior to NGA OAPs in reducing hospitalization rates [15]. In cohort studies, however, time order effects, prescribing bias and lack of blinding. may affect outcomes [6••, 10]. Moreover, no reverse mirror image studies such as switching from LAIs to OAPs have been conducted [16].

Each of the study designs has methodological limitations; thus, further prospective longer term large pragmatic trials designed to reflect everyday clinical practice are necessary to evaluate the efficacy of LAIs versus OAPs.

## Safety and tolerability of LAIs versus oral antipsychotics

In a recent meta-analysis of 16 RCTs comparing the same antipsychotics, LAIs and OAPs did not significantly differ with respect to the frequency of treatment discontinuation due to adverse events, serious adverse events, or all-cause death [17•]. There were also no differences between LAIs and OAPs in extrapyramidal symptoms (EPSs) except for akinesia, as well as for weight gain and all metabolic adverse events except for LDL-cholesterol. However, another meta-analysis of NGA LAIs showed a greater risk of EPS with NGA LAIs than with OAPs [5]. NGA LAIs and OAPs were associated with similar risks of metabolic adverse events, suggesting that pharmacokinetic differences between the two preparations are irrelevant to this adverse event profile [18]. On the other hand, LAIs showed a significantly lower change in serum prolactin levels compared with OAPs [17•]. In another recent meta-analysis of 52 RCTs, there was no significant difference between LAIs and OAPs with respect to all-cause death and death due to suicide [19].

Compared with OAPs, the pharmacokinetics of LAIs such as the long elimination half-life can delay both the onset and the remission of adverse effects [18]. However, few studies have compared NGA LAIs with OAPs in terms of adverse event severity and time course. Moreover, studies on adverse events for NGA LAIs after the discontinuation of supplemental oral antipsychotics are required [17•].

## Efficacy of LAIs versus LAIs

Only a few direct comparisons between one LAI and another have been conducted [20–23, 24•]. These indicate that relapse rates and symptom improvement are similar with different LAIs including FGA LAIs and NGA LAIs [6••]. At present, there appears to be no advantage of one LAI over another in general efficacy. Differences among LAIs were observed in some other efficacy and safety domains as described below. Further prospective large head-to-head comparison studies of different LAIs are warranted.

## Specific agents

### Risperidone LAI

Risperidone LAI (RLAI) was introduced as the first NGA LAI in 2003. It is a microsphere preparation, and biweekly dosing intervals are needed [4]. Oral supplementation at the time of starting is required for 3 weeks. Its approval was based on three pivotal trials [25–27], which demonstrated advantages over placebo, comparable efficacy to oral risperidone, and acceptable long-term safety. It has been widely used since. It failed to differentiate from psychiatrists’ choice of oral antipsychotics in a large-scale single-blind comparative study conducted in the US Veterans Administration system regarding hospitalization rates [28].

On the other hand, in the previously cited cohort study in Finland, the authors found a significantly lower risk of rehospitalization on depots than on oral drugs [13], while in yet another nationwide retrospective inception cohort study in Denmark, RLAI and FGA LAIs did not differ regarding time to hospitalization, all-cause discontinuation, or duration of hospitalization in patients with schizophrenia [23]. However, this study did not evaluate psychopathology, functional outcomes, quality of life (QOL), or side effects.

### Olanzapine pamoate

Olanzapine pamoate LAI (OLAI) received marketing approval in 2008 in the EU and in 2009 in the USA [4]. It is a crystalline salt formulation consisting of olanzapine and pamoic acid [29]. Supplemental oral olanzapine is not necessary at the initiation of OLAI [4].

In a 24-week double-blind RCT comparing OLAI with oral olanzapine in stable outpatients with schizophrenia, OLAI showed comparable efficacy and safety to oral olanzapine, except for injection-related adverse events [30]. OLAI can induce post-injection delirium/sedation syndrome (PDSS) in a small proportion of patients [29]. PDSS occurred in approximately 0.07% of injections and in 1.03% of patients in routine clinical practice [31]. So far, there were no fatalities by PDSS, and most cases resolved within 72 h [18]. OLAI must be administered in registered healthcare facilities, and all patients should be monitored for at least 3 h after injection [29]. To date, studies to compare OLAI with other LAIs or other oral antipsychotics are lacking.

### Once-monthly paliperidone palmitate

Paliperidone palmitate (PP), a crystalline composition of 9-hydroxyrisperidone, is the first once-monthly NGA LAI available, and supplementation with oral paliperidone is not required after the injection. [4]. It was approved for use in 2009 in the USA and the EU.

Several double-blind RCTs were conducted to compare the effectiveness of PP with other LAIs, including RLAI [20, 32–35] and haloperidol decanoate (HPD) [22], and indirectly compared to oral paliperidone extended release (ER) with respect to tardive dyskinesia risk [36]. In general, the results demonstrated similar efficacy and safety outcomes when comparing PP to RLAI or oral paliperidone ER in patients with recently diagnosed schizophrenia, adult patients with chronic schizophrenia [20, 32, 36], and patients with markedly to severely ill schizophrenia [34]. However, in a 53-week RCT, PP was not as efficacious as RLAI, which may have been due to an inadequate initial dosing strategy, since modified [33]. In patients with schizophrenia or schizoaffective disorder, PP was not superior to HPD in terms of prevention of efficacy failure [22] although this study was hampered by a considerable dropout rate [37]. Moreover, PP was associated with more weight gain and higher prolactin levels but showed a lower propensity to cause EPS than HPD.

### Three-monthly paliperidone palmitate

Three-monthly paliperidone palmitate (3MPP) was approved for marketing in 2015 in the USA. It was superior to placebo in a relapse prevention study [38]. In a 48-week double-blind non-inferiority RCT, 3MPP showed similar relapse rates compared with monthly PP (8 versus 9%) in patients with schizophrenia who were previously stabilized with monthly PP [39]. The safety and tolerability profiles of the two formulations were also similar. The most common treatment-emergent adverse event was weight gain.

### Aripiprazole LAI

Aripiprazole LAI (ALAI) was approved for use in 2013. It is a lyophilized powder of aripiprazole, and oral antipsychotic supplementation is recommended for 2 weeks after the first injection [29].

Its efficacy against relapses was demonstrated in a placebo-controlled 6-month trial [40]. In two double-blind RCTs comparing ALAI with oral aripiprazole, ALAI was non-inferior to oral aripiprazole in patients with schizophrenia with respect to relapse risk and showed a comparable safety profile [41, 42]. In a phase IIIb, 28-week, open-label, head-to-head RCT, ALAI showed superior improvements over PP on health-related QOL and psychosocial functioning [24•]. Moreover, ALAI demonstrated a more favorable safety profile compared with PP. This is the first evidence to show superiority of one NGA LAI over another.

Aripiprazole lauroxil became available in 2015 in the USA. It can be initiated in three different dosages and has the option of 6-week dosing [43]. Oral aripiprazole supplementation is necessary for 3 weeks. It also showed robust efficacy for treating acute exacerbations of schizophrenia [44].

### LAI antipsychotics in early stages of schizophrenia

The first 2–5 years after the onset of schizophrenia are considered to be critical in determining long-term prognosis [45]. Poor adherence to oral medication is thought to be the most frequent cause of relapse [46]. LAI antipsychotics may have a benefit in terms of managing adherence, thereby reducing relapse risk during early stages of the illness or in first-episode patients, a population that frequently shows non-adherence and relapses [6••, 47•].

In an open-label RCT comparing the effectiveness of RLAI with oral NGAs in patients during the early phase of a schizophrenia spectrum disorder, no differences were observed for psychotic symptom control [48]. Weiden et al. [49, 50] conducted a RCT in patients with first-episode schizophrenia and also found no difference in adherence between RLAI and OAPs. However, in a recent RCT comparing the effectiveness of RLAI with oral risperidone in patients with first-episode schizophrenia, RLAI showed better adherence, greater relapse prevention, and better symptom control than oral risperidone [51•]. Similarly, Schreiner et al. [52] compared once-monthly PP to various oral NGAs in a randomized open-label trial in recently diagnosed schizophrenia patients and found advantages of PP in terms of relapse prevention.

Evidence for other NGA LAIs is limited, and the proposal to recommend LAIs to patients with early stages or first-episode schizophrenia still debated [53–55]. When an LAI is administered for a patient with first-episode schizophrenia, safety and tolerability should firstly be evaluated with the oral equivalent and the LAI should be initiated at a low dose [18]. Further studies examining the effects of early intervention with NGA LAIs on long-term outcome including cost-effectiveness are needed.

## Discussion

Several NGAs have become available as long-acting formulations. Undoubtedly, NGA LAIs have extended the range of long-term therapeutic options for patients with schizophrenia. NGA LAIs appear not to confer a greater risk of serious adverse events, compared with OAPs [17•]. It is important to note that NGA LAIs have different characteristics in terms of pharmacologic and pharmacokinetic profiles, cost, injection sites, needle gauge, injection volume, injection interval, dosage variation, storage needs, need for oral supplementation, observation period following injection, potential drug interactions, and adverse events [6••, 29, 54]. When using classical measures of psychopathology as endpoints, head-to-head studies have mostly demonstrated non-inferiority between different LAIs [20, 21]. Thus, when choosing an LAI, clinicians need to primarily consider side effect liability of the respective LAI. For example, potential concerns about the use of RLAI and PP are their propensity to cause EPS and hyperprolactinemia. OLAI has liability to induce weight gain, metabolic adverse events, and PDSS. ALAI appears to have advantages in terms of being essentially free of prolactin elevation or significant metabolic side effects [29]. For clinicians, the appreciation of these differences is useful for selecting LAIs for the treatment of individual patients, especially also considering treatment history [6••, 18].

Formulations with a longer dosing interval offer patients convenience regarding the frequency of injections and provide clinicians more time to intervene in case of adherence problems. However, Olfson et al. [56] reported that early discontinuation of LAIs is quite common in the community treatment of schizophrenia. A great majority of patients starting LAIs discontinue use within the first few months of treatment. Thus, assuring a reliable therapeutic relationship between mental health professionals and patients through regular contact is particularly relevant for the successful treatment with LAIs on a 3-month schedule [38, 57•].

There are significant differences in LAI use across regions, countries, and over the years. Despite the available encouraging evidence, LAIs, at present, are not widely prescribed for a variety of reasons, including the reservations of patients, clinicians, and payers [57•]. This is especially significant for patients in the early course of schizophrenia, where there are pro and con arguments for the use of LAIs [53–55]. Given the significance of continuous therapy in this critical period, physicians should consider all available treatment options, and the risk-benefit balance of LAI use needs to be thoroughly assessed for individual patients in a shared decision-making approach [47•, 54]. Adequate education of mental health staff, patients, and families regarding the benefits and limitations of LAIs may reduce the negative image and prejudice towards the use of LAIs and increase acceptance within a strong therapeutic alliance [6••, 47•, 57•, 58, 59].

## Conclusion

Recently introduced NGA LAIs broaden the options for the treatment of schizophrenia. However, current evidence regarding the effectiveness of NGA LAIs is still limited. For example, the question of whether NGA LAIs have advantages over FGA LAIs and/or oral antipsychotics is still open. Further head-to-head clinical trials comparing different LAIs in terms of efficacy, safety, functioning, and QOL under real-life treatment conditions are warranted to clarify the role of the individual LAIs in the long-term treatment of schizophrenia.

## References

[1] Miyamoto S, Fleischhacker WW, Lieberman JA, Lieberman JA, Murray R (2012). Pharmacologic treatment of schizophrenia. Comprehensive Care of Schizophrenia (Second Edition): A Textbook of Clinical Management.

[2] Fleischhacker WW, Miyamoto S (2016). Pharmacological treatment of schizophrenia: current issues and future perspectives. Clinical Neuropsychopharmacology and Therapeutics.

[3] Fleischhacker WW (2009). Second-generation antipsychotic long-acting injections: systematic review. Br J Psychiatry Suppl..

[4] Rauch AS, Fleischhacker WW (2013). Long-acting injectable formulations of new-generation antipsychotics: a review from a clinical perspective. CNS Drugs.

[5] Fusar-Poli P, Kempton MJ, Rosenheck RA (2013). Efficacy and safety of second-generation long-acting injections in schizophrenia: a meta-analysis of randomized-controlled trials. Int Clin Psychopharmacol.

[6] Correll CU, Citrome L, Haddad PM, Lauriello J, Olfson M, Calloway SM (2016). The use of long-acting injectable antipsychotics in schizophrenia: evaluating the evidence. J Clin Psychiatry.

[7] Kishimoto T, Robenzadeh A, Leucht C, Leucht S, Watanabe K, Mimura M (2014). Long-acting injectable vs oral antipsychotics for relapse prevention in schizophrenia: a meta-analysis of randomized trials. Schizophr Bull.

[8] Leucht C, Heres S, Kane JM, Kissling W, Davis JM, Leucht S (2011). Oral versus depot antipsychotic drugs for schizophrenia—a critical systematic review and meta-analysis of randomised long-term trials. Schizophr Res.

[9] Adams CE, Fenton MK, Quraishi S, David AS (2001). Systematic meta-review of depot antipsychotic drugs for people with schizophrenia. Br J Psychiatry.

[10] Haddad PM, Kishimoto T, Correll CU, Kane JM (2015). Ambiguous findings concerning potential advantages of depot antipsychotics: in search of clinical relevance. Curr Opin Psychiatry.

[11] Kishimoto T, Nitta M, Borenstein M, Kane JM, Correll CU (2013). Long-acting injectable versus oral antipsychotics in schizophrenia: a systematic review and meta-analysis of mirror-image studies. J Clin Psychiatry..

[12] Kirson NY, Weiden PJ, Yermakov S, Huang W, Samuelson T, Offord SJ (2013). Efficacy and effectiveness of depot versus oral antipsychotics in schizophrenia: synthesizing results across different research designs. J Clin Psychiatry..

[13] Tiihonen J, Haukka J, Taylor M, Haddad PM, Patel MX, Korhonen P (2011). A nationwide chohort study of oral and depot antipsychotics after first hospitalization for schizophrenia. Am J Psychiatry.

[14] Bitter I, Katona L, Zámbori J, Takács P, Fehér L, Diels J (2013). Comparative effectiveness of depot and oral second generation antipsychotic drugs in schizophrenia: a nationwide study in Hungary. Eur Neuropsychopharmacol.

[15] Lafeuille MH, Dean J, Carter V, Duh MS, Fastenau J, Dirani R (2014). Systematic review of long-acting injectables versus oral atypical antipsychotics on hospitalization in schizophrenia. Curr Med Res Opin.

[16] Montemagni C, Frieri T, Rocca P (2016). Second-generation long-acting injectable antipsychotics in schizophrenia: patient functioning and quality of life. Neuropsychiatr Dis Treat.

[17] Misawa F, Kishimoto T, Hagi K, Kane JM, Correll CU (2016). Safety and tolerability of long-acting injectable versus oral antipsychotics: a meta-analysis of randomized controlled studies comparing the same antipsychotics. Schizophr Res.

[18] Haddad P, Fleischhacker WW. Adverse effects and antipsychotic long-acting injections. In: Haddad P, Lambert T, Lauriello J, editors. Antipsychotic long-acting injections, Second edition. Oxford University Press 2016. p. 59–85.

[19] Kishi T, Matsunaga S, Iwata N (2016). Mortality risk associated with long-acting injectable antipsychotics: a systematic review and meta-analyses of randomized controlled trials. Schizophr Bull.

[20] Pandina G, Lane R, Gopal S, Gassmann-Mayer C, Hough D, Remmerie B (2011). A double-blind study of paliperidone palmitate and risperidone long-acting injectable in adults with schizophrenia. Prog Neuro-Psychopharmacol Biol Psychiatry.

[21] Li H, Rui Q, Ning X, Xu H, Gu N (2011). A comparative study of paliperidone palmitate and risperidone long-acting injectable therapy in schizophrenia. Prog Neuro-Psychopharmacol Biol Psychiatry.

[22] McEvoy JP, Byerly M, Hamer RM, Dominik R, Swartz MS, Rosenheck RA (2014). Effectiveness of paliperidone palmitate vs haloperidol decanoate for maintenance treatment of schizophrenia: a randomized clinical trial. JAMA.

[23] Nielsen J, Jensen SO, Friis RB, Valentin JB, Correll CU (2015). Comparative effectiveness of risperidone long-acting injectable vs first-generation antipsychotic long-acting injectables in schizophrenia: results from a nationwide, retrospective inception cohort study. Schizophr Bull.

[24] Naber D, Hansen K, Forray C, Baker RA, Sapin C, Beillat M (2015). Qualify: a randomized head-to-head study of aripiprazole once-monthly and paliperidone palmitate in the treatment of schizophrenia. Schizophr Res.

[25] Kane JM, Eerdekens M, Lindenmayer JP, Keith SJ, Lesem M, Karcher K (2003). Long-acting injectable risperidone: efficacy and safety of the first long-acting atypical antipsychotic. Am J Psychiatry.

[26] Fleischhacker WW, Eerdekens M, Karcher K, Remington G, Llorca PM, Chrzanowski W (2003). Treatment of schizophrenia with long-acting injectable risperidone: a 12-month open-label trial of the first long-acting second-generation antipsychotic. J Clin Psychiatry..

[27] Chue P, Eerdekens M, Augustyns I, Lachaux B, Molcan P, Eriksson L (2005). Comparative efficacy and safety of long-acting risperidone and risperidone oral tablets. Eur Neuropsychopharmacol.

[28] Rosenheck RA, Krystal JH, Lew R, Barnett PG, Fiore L, Valley D (2011). Long-acting risperidone and oral antipsychotics in unstable schizophrenia. N Engl J Med.

[29] Citrome L (2013). New second-generation long-acting injectable antipsychotics for the treatment of schizophrenia. Expert Rev Neurother.

[30] Kane JM, Detke HC, Naber D, Sethuraman G, Lin DY, Bergstrom RF (2010). Olanzapine long-acting injection: a 24-week, randomized, double-blind trial of maintenance treatment in patients with schizophrenia. Am J Psychiatry.

[31] Bushe CJ, Falk D, Anand E, Casillas M, Perrin E, Chhabra-Khanna R (2015). Olanzapine long-acting injection: a review of first experiences of post-injection delirium/sedation syndrome in routine clinical practice. BMC Psychiatry.

[32] Alphs L, Bossie CA, Sliwa JK, Fu DJ, Ma YW, Hulihan J (2013). Paliperidone palmitate and risperidone long-acting injectable in subjects with schizophrenia recently treated with oral risperidone or other oral antipsychotics. Neuropsychiatr Dis Treat.

[33] Fleischhacker WW, Gopal S, Lane R, Gassmann-Mayer C, Lim P, Hough D (2012). A randomized trial of paliperidone palmitate and risperidone long-acting injectable in schizophrenia. Int J Neuropsychopharmacol.

[34] Fu DJ, Bossie CA, Kern Sliwa J, Ma YW, Alphs L (2014). Paliperidone palmitate versus risperidone long-acting injection in markedly-to-severely ill schizophrenia subjects: onset of efficacy with recommended initiation regimens. Clin Schizophr Relat Psychoses.

[35] Fu DJ, Bossie CA, Sliwa JK, Ma YW, Alphs L (2014). Paliperidone palmitate versus oral risperidone and risperidone long-acting injection in patients with recently diagnosed schizophrenia: a tolerability and efficacy comparison. Int Clin Psychopharmacol.

[36] Gopal S, Xu H, Bossie C, Buron JA, Fu DJ, Savitz A (2014). Incidence of tardive dyskinesia: a comparison of long-acting injectable and oral paliperidone clinical trial databases. Int J Clin Pract.

[37] Fleischhacker WW (2014). Antipsychotic medications for schizophrenia. JAMA.

[38] Berwaerts J, Liu Y, Gopal S, Nuamah I, Xu H, Savitz A (2015). Efficacy and safety of the 3-month formulation of paliperidone palmitate vs placebo for relapse prevention of schizophrenia: a randomized clinical trial. JAMA psychiatry.

[39] Savitz AJ, Xu H, Gopal S, Nuamah I, Ravenstijn P, Janik A, et al. Efficacy and safety of paliperidone palmitate 3-month formulation for patients with schizophrenia: a randomized, multicenter, double-blind, noninferiority study. Int J Neuropsychopharmacol. 2016;19(7) doi:10.1093/ijnp/pyw018. 10.1093/ijnp/pyw018 PMC4966278 26902950

[40] Kane JM, Sanchez R, Perry PP, Jin N, Johnson BR, Forbes RA (2012). Aripirazole intramuscular depot as maintenance treatment in patients with schizophrenia: a 52-week, multicenter, randomized, double-blind, placebo-controlled study. J Clin Psychiatry..

[41] Fleischhacker WW, Sanchez R, Perry PP, Jin N, Peters-Strickland T, Johnson BR (2014). Aripiprazole once-monthly for treatment of schizophrenia: double-blind, randomised, non-inferiority study. Br J Psychiatry.

[42] Ishigooka J, Nakamura J, Fujii Y, Iwata N, Kishimoto T, Iyo M (2015). Efficacy and safety of aripiprazole once-monthly in Asian patients with schizophrenia: a multicenter, randomized, double-blind, non-inferiority study versus oral aripiprazole. Schizophr Res.

[43] Aggarwal A, Gopalakrishna G, Lauriello J (2016). Aripiprazole lauroxil long-acting injectable: the latest addition to second-generation long-acting agents. Clin Schizophr Relat Psychoses..

[44] Meltzer HY, Risinger R, Nasrallah HA, Du Y, Zummo J, Corey L (2015). A randomized, double-blind, placebo-controlled trial of aripiprazole lauroxil in acute exacerbation of schizophrenia. J Clin Psychiatry..

[45] Birchwood M, Todd P, Jackson C (1998). Early intervention in psychosis. The critical period hypothesis. Br J Psychiatry Suppl..

[46] Lindenmayer JP, Liu-Seifert H, Kulkarni PM, Kinon BJ, Stauffer V, Edwards SE (2009). Medication nonadherence and treatment outcome in patients with schizophrenia or schizoaffective disorder with suboptimal prior response. J Clin Psychiatry..

[47] Brissos S, Veguilla MR, Taylor D, Balanza-Martinez V (2014). The role of long-acting injectable antipsychotics in schizophrenia: a critical appraisal. Therapeutic advances in psychopharmacology.

[48] Malla A, Chue P, Jordan G, Stip E, Koczerginski D, Milliken H (2016). An exploratory, open-label, randomized trial comparing risperidone long-acting injectable with oral antipsychotic medication in the treatment of early psychosis. Clin Schizophr Relat Psychoses.

[49] Weiden PJ, Schooler NR, Weedon JC, Elmouchtari A, Sunakawa-McMillan A (2012). Maintenance treatment with long-acting injectable risperidone in first-episode schizophrenia: a randomized effectiveness study. J Clin Psychiatry..

[50] Weiden PJ, Schooler NR, Weedon JC, Elmouchtari A, Sunakawa A, Goldfinger SM (2009). A randomized controlled trial of long-acting injectable risperidone vs continuation on oral atypical antipsychotics for first-episode schizophrenia patients: initial adherence outcome. J Clin Psychiatry.

[51] Subotnik KL, Casaus LR, Ventura J, Luo JS, Hellemann GS, Gretchen-Doorly D (2015). Long-acting injectable risperidone for relapse prevention and control of breakthrough symptoms after a recent first episode of schizophrenia. A randomized clinical trial. JAMA psychiatry.

[52] Schreiner A, Aadamsoo K, Altamura AC, Franco M, Gorwood P, Neznanov NG (2015). Paliperidone palmitate versus oral antipsychotics in recently diagnosed schizophrenia. Schizophr Res.

[53] Kim B, Lee SH, Yang YK, Park JI, Chung YC (2012). Long-acting injectable antipsychotics for first-episode schizophrenia: the pros and cons. Schizophrenia research and treatment.

[54] Stevens GL, Dawson G, Zummo J (2016). Clinical benefits and impact of early use of long-acting injectable antipsychotics for schizophrenia. Early intervention in psychiatry..

[55] Emsley R, Chiliza B, Asmal L, Mashile M, Fusar-Poli P (2013). Long-acting injectable antipsychotics in early psychosis: a literature review. Early intervention in psychiatry.

[56] Olfson M, Marcus SC, Ascher-Svanum H (2007). Treatment of schizophrenia with long-acting fluphenazine, haloperidol, or risperidone. Schizophr Bull.

[57] Parellada E, Bioque M (2016). Barriers to the use of long-acting injectable antipsychotics in the management of schizophrenia. CNS Drugs.

[58] Waddell L, Taylor M (2009). Attitudes of patients and mental health staff to antipsychotic long-acting injections: systematic review. Br J Psychiatry Suppl.

[59] Kirschner M, Theodoridou A, Fusar-Poli P, Kaiser S, Jager M (2013). Patients’ and clinicians’ attitude towards long-acting depot antipsychotics in subjects with a first episode of psychosis. Therapeutic advances in psychopharmacology.

